# Streamlined data extraction and visualization utilizing third‐party verification software for online adaptive radiation therapy

**DOI:** 10.1002/acm2.70397

**Published:** 2025-12-26

**Authors:** Sean Tanny, Alexander Podgorsak, Nicholas Sperling

**Affiliations:** ^1^ Department of Radiation Oncology University of Rochester Medical Center New York New York USA; ^2^ Department of Radiation Oncology University of Toledo Medical Center Toledo Ohio USA

**Keywords:** adaptive radiotherapy, CBCT‐based adaptive RT, Ethos‐based adaptive

## Abstract

**Purpose:**

Online adaptive radiation therapy (oART) has generated a significant influx of daily treatment planning data. Present oART platforms have leveraged automation for decreased planning times at the expense of end‐user access, making longitudinal oART data assessment time consuming. We have developed modified software that extracts oART data from the secondary calculation software associated with one of the oART platforms.

**Methods:**

A Python program was developed to query oART data from a web‐based secondary dose calculation software. Structure, plan, and dosimetric data were collected for all generated oART plans over the course of 3 years. This software stores data in a Pandas dataframe to allow for comparisons and advanced visualization. The software includes some pre‐defined visualization and analysis methods, as well as tools to de‐identify and share cohorts of oART data.

**Results:**

Twelve hundred oART sessions were pulled from our secondary dose verification software. Various visualization methods were implemented and are demonstrated in this technical note. This software demonstrated significant time savings in data extraction compared to manual session exports, but does not pull raw DICOM information, such as CT scans, structure sets, or dose files.

**Conclusion:**

Our extraction software presents a streamlined way to collect, analyze, and visualize oART data using a dedicated oART platform. Further tools are needed to improve access and analysis of oART data.

## 1 INTRODUCTION

Online adaptive radiation therapy is one of many cutting‐edge techniques that offers dosimetric advantages over traditional image‐guided radiation therapy (IGRT) techniques by adapting a pre‐existing treatment plan to daily variations in the patient's anatomy.^1^ Release and rapid adoption of CBCT‐guided adaptive radiotherapy (ART) platforms has created a surge of interest in assessing how well oART accomplishes the goal of adapting to daily variations. The initial implementation of the Varian Ethos ART platform (Varian Medical Systems, Inc., Palo Alto, CA) restricted users from accessing much of the data the system produces unless the treatment is manually exported—a time intensive process for a busy clinic. The recent release of Ethos V2.0 improves R&V connectivity and DICOM data accessibility, but only for patients treated using IGRT. Access to oART treatment data remains a manual export from the Ethos TPMS. Data accessibility may hinder clinical research efforts to characterize Ethos system performance relative to IGRT, particularly when the number of patients treated worldwide continues to scale with increased oART use.

The Ethos system was designed to optimize the efficiency of the on‐couch treatment session, evinced in design decisions which limit the number of DICOM targets the system will accept to a single source for on‐couch sessions—a dedicated, pre‐packaged solution for fast secondary verification based on the Mobius3D (Varian Medical System, Inc., Palo Alto, CA) system. The Ethos platform sends DICOM RT Plan, RT Structure, RT Dose, and CT information to Mobius, where that data are used to validate contour integrity, dose calculation accuracy, and adherence to departmental dose constraints. The Mobius system also receives treatment log‐files after treatment delivery as a method for verifying the treatment delivery integrity.

Mobius provides an API to permit users to query the database and extract some of the planning and dosimetric data, as an alternative to manually retrieving the data from the Ethos system. We have developed a tool that extracts plan, structure, and dosimetry data and stores this data in an accessible format for analysis of oART treatments using the Varian Ethos oART platform. We have also developed some basic visualization tools for plotting dose–volume histograms and longitudinal volume comparisons per patient.

## 2 METHODS

The MobiusEthosDataExtractor module (MEDE) includes utilities for both the collection of data from the Ethos‐dedicated Mobius system, as well as tools to process and analyze that data.

### 2.1 Mobius data structure

Mobius3D is a relational database sold with the Varian Ethos adaptive radiotherapy platform paired to work specifically with the Ethos system. Mobius performs both pretreatment plan calculations and posttreatment log‐file analysis to verify treatment delivery accuracy. This version of Mobius uses Check IDs as a primary key. Each check is accessible through a web‐request session in JSON format. We can utilize this structure to scrape pretreatment plan checks for a list of oART patients and determine which plans were delivered on the treatment system based on the existence of a linked log‐file check ID.

The Mobius system stores DVH data for each structure from both the TPMS or on‐couch session and from the Mobius secondary dose calculation and reconstructions. Mobius also stores some beam information, including isocenter coordinates, number of control points, gantry and collimator angles, and MU per beam which can be used to calculate the modulation factor.^2^

### 2.2 Ethos Data Generation and Labeling

The Ethos system creates an initial structure set for each session based on the influencer contouring and deformed targets generated by the users at the beginning of the adaptive session. Modifying any of these contours after target deformation from the reference CT to the daily synthetic CT (sCT) will create a new structure set and pair of online adaptive plans. These plans are named in the format “**[Reference Plan ID]/[SCH/ADP][2 Digit Fraction Number][2 digit revision number]**”. For example, the default pair of daily sessions for a plan with reference plan ID IM103 on fraction 14 would be named IM103/SCH1401 and IM103/ADP1402. A subsequent revision of the structure set would produce the pair of plans IM103/SCH1403 and IM103/ADP1404. The last two digits could be exchanged (e.g., ADP1403 and ADP1404 are equally valid identifiers), but will iterate by two with each structure set revision. We utilize the “planName_str” field to identify several components of the plan: plan type (Reference, Scheduled, or Adaptive), fraction number, revision number of the structure set (e.g., ADP1403 would be revision 2), and treatment site as defined by the planning directive.

The MEDE comes with a pre‐defined list of organs at risk (OARs) based on the pre‐existing structures within the Ethos planning directives pertinent to each site as well as some structures utilized at our institution. These are defined in the function “set_oars” that allows the user to define the relevant OARs for various treatment sites. These are grouped into related treatment sites, such as pelvic, thoracic, and head and neck regions, but could be separated out as more institution specific protocols. Mobius contains information regarding the structure volume and any relevant density overrides. MEDE will then compute structure volume differences relative to the reference structure set.

### 2.3 Data collection

The data are pulled from the Mobius database using the API provided in the Mobius Instruction for Use documentation under the section for Raw Data Access. A Python based software tool previously released by Varian was modified and extended to collect the desired data.

**FIGURE 1:**
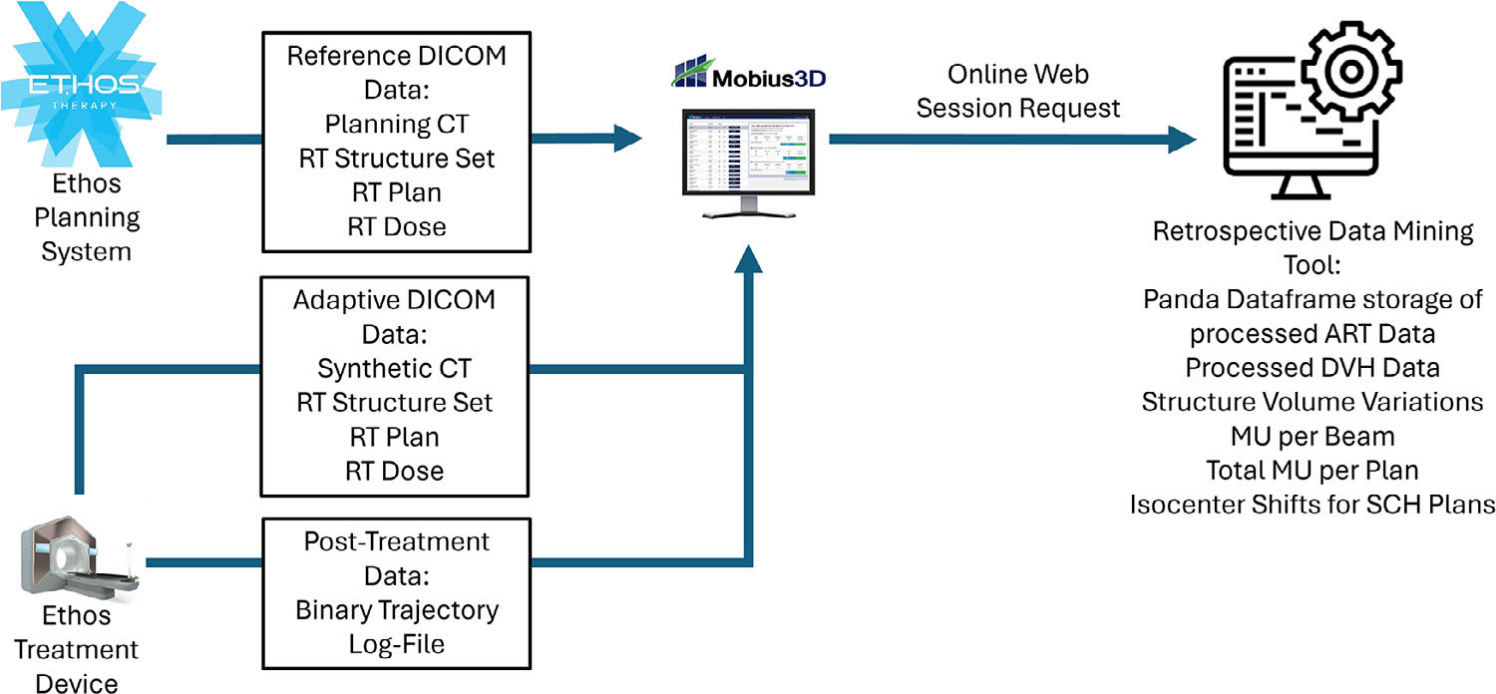
Data workflow diagram demonstrating the flow of data from the treatment device to the secondary dose calculation software and subsequent scripted processing by MEDE.

MEDE uses a list of patient IDs to create a request from the Mobius database for the list of plan checks stored for each patient. These checks build a list of all plans developed or treated per patient on the Ethos system. The tool will determine which type of plan each plan check was and will link together plan lists to determine which plan was treated for each fraction. After building a list of plans to be collected, the tool will pull the data received from the Ethos system as described in Section 2b. The MEDE code will then pass the data to the analysis and storage methods. This workflow is presented in Figure 1.

### 2.4 Data analysis and storage model

This tool calculates some basic plan data and DVH metrics using the Ethos data that has been resampled in Mobius to be stored for direct access in a Pandas dataframe^3^ as well as a representation of the DVH data which may be used to calculate arbitrary metrics for each structure as the need may arise.

The MEDE tool will compile relevant OARs and identify target structures based on ROI names, assigning them to PTVLow, PTVInt, etc. The tool will also collect dose details for structures, information about each plan such as treated status, adapted status, revision number, and some details of the plan which may be used to evaluate simple plan complexity metrics. As the DICOM plans are not accessible from the Mobius API, beam information such as MLC positions for each control point are not available, and so other measures of plan complexity which would rely on this data are unable to be calculated.

Upon collecting the dataset for each patient, a Python “pickle” containing the full patient dataset is saved, and the collected patient dataset are added to the full set for all patients. In addition, each patient is assigned a unique sequential ID in the database based on the user defined institution ID and index in the database. This allows for potential for institutions to deidentify data while maintaining the institution's ability to append data for distinct patients.

Finally, the collected dataset are connected through a datacollector class object which unifies methods of storing the data to allow multiple different storage solutions. A virtual class has been implemented which may be subclassed, as well as a “pickle_datacollector” class which allows the datacollector to be stored in a standard python pickle.

### 2.5 Collaboration features

The use of a site‐unique prefix and dataset identifier permits the possibility of collaborative data exchange with other institutions, if approved under the supervision of an appropriate IRB in accordance with each institution's investigational protocols. Additionally, the use of pandas as the data store, and the introduction of the “pickle_datacollector” class permits the export of segments of the dataset to a compact compressed file format. This data may then be brought into multiple institutions to allow comparison between institutions and the development of collaborative protocols for adaptive therapy.

The code is written to be extensible and interoperable with additional data columns between institutions. This will allow for institutions to develop additional analysis functions which can be stored in the dataset and shared with outside groups without causing compatibility issues beyond potential namespace collisions for the columns.

Finally, the code base for this work is licensed under Creative Commons Attribution‐ShareAlike 4.0 International. The code for this work is publicly available on GitHub. The code is available at https://github.com/seantanny/MobiusEthosDataExtractor.

## 3 RESULTS

### 3.1 Data extraction time and size

Export times for individual sessions ranged from 228 – 391 seconds from the Ethos TPMS over 22 sessions, with a mean session export time of 5 min. This would correspond to a total time of 190 min to export a full course of adaptive treatments for a 38‐fraction patient, neglecting any user delay or interface time. Our data extractor was able to extract and analyze all treatments from 50 adaptive patients in 140 min.

### 3.2 Data visualization examples

A set of functions and examples were developed to aid in the visualization of the resulting data collections. Included are tools to present aggregate DVH data as a DVH sliding box and whiskers plot, or DVH spread plot, for structures.

A module for data visualization is available as an import. Accepts a dataframe as a source and will provide visualization functions for individual patients, or tools for longitudinal examination across the entire patient set. By using pandas dataframes as a source, all the filtering tools available in a dataframe are available to use for stratification in the longitudinal assessment tools.

### 3.3 Longitudinal assessment of OAR variations

**FIGURE 2:**
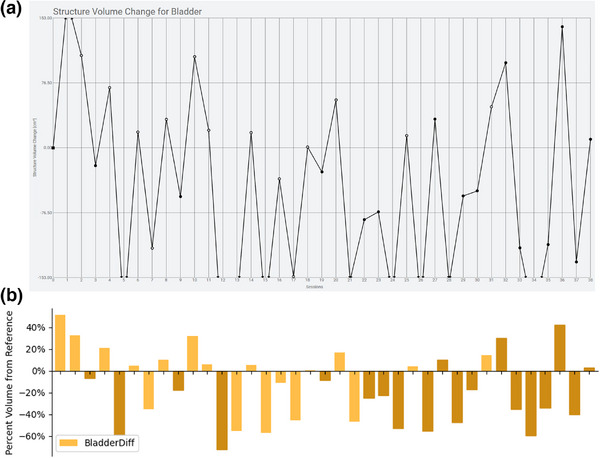
Comparison between (a) the volume trending visualization in the Ethos TPMS and (b) the trending visualized using the MEDE toolset for the same patient. Relative and absolute visualizations can be achieved, as well as color coding which sessions were adapted versus treated with the scheduled plan.

MEDE is designed to assess variations in volumes and plan parameters relative to the reference volumes and plan. Our initial dataset has extracted 19 patients treated with oART over 604 adaptive sessions. All of these patients were treated to their pelvic regions, with primary OARs being bladder, rectum, and bowelspace. Longitudinal assessments of volume changes over the course of oART for two representative patients are presented in Figure 2. Changes in bladder volume over the course of therapy are shown relative to the plot available within the Ethos Treatment Planning Management System (TPMS) in the Monitoring workspace. The data presented within the Ethos TPMS are neither tabular nor able to be easily captured by clinicians. This demonstrates that the MEDE accurately assesses volume changes relative to baseline, can visualize the entire range of variation, and present the data in various formats that may be helpful for clinicians.

### 3.4 DVH visualization

**FIGURE 3:**
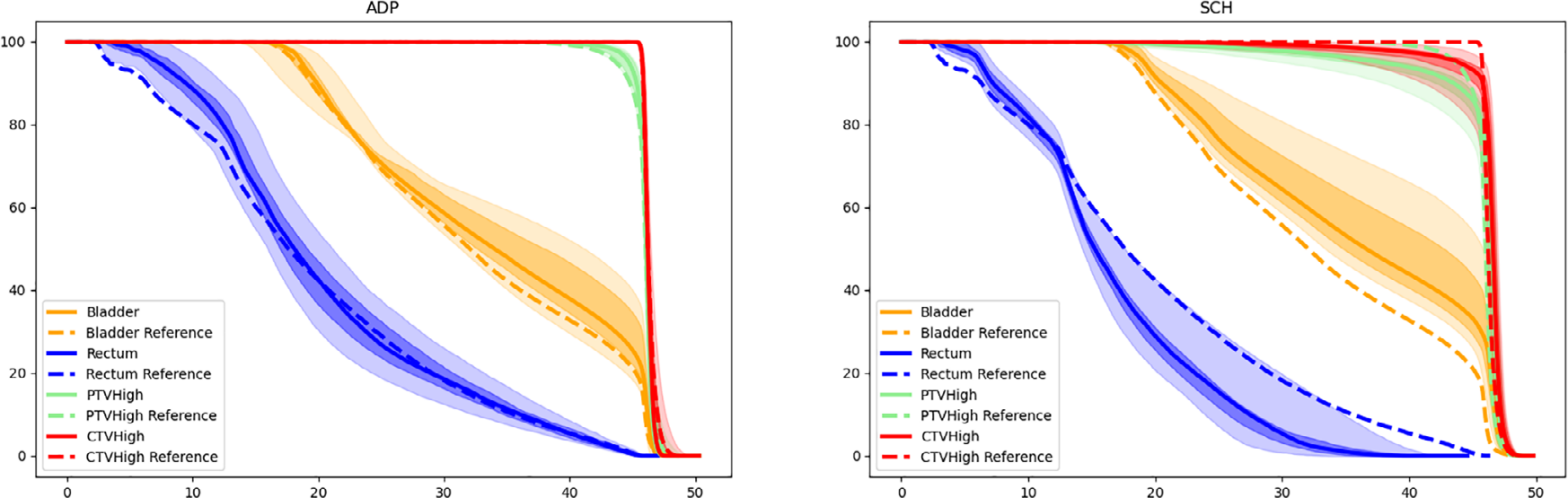
DVH Visualization from the MEDE dataset over the primary course of adaptive therapy for a 25 fraction treatment to the prostatic fossa and pelvic lymph nodes. PTV, CTV, bladder, and rectum DVH curves are presented. The light shaded regions represent the extrema of the DVH data, the darker shaded region is the 20^th^–80^th^ percentile range from daily treatment DVH data, the solid line is the median DVH data, and the dashed line is the reference DVH curve for each structure. DVH data from adaptive plans are on the left and scheduled plans are on the right.

MEDE DVH visualization and validation is demonstrated in Figure 3. Visualization is shown using the total range of DVHs, median DVH curve for each OAR, 20^th^ and 80^th^ percentiles of each structure's DVH over the course of adaptive therapy per treatment phase (primary vs. boost), and the reference DVH curve as a dashed line. DVH data were first converted from a truncated list (as described in Section 2.a) into an interpolation function that was padded to be 100% or 0% if the entered dose value was either below or above the list contents, respectively. This allows for more accurate percentile reporting over the range of treatments. Figure 3 does not present any individual DVH curve from a specified treatment, although that data could be plotted. It is instead more of a sliding box and whiskers plot representing the DVH distribution obtained from the various scheduled and adaptive plans generated from the patient's treatment course.

### 3.5 Dosimetric objective distributions

**FIGURE 4:**
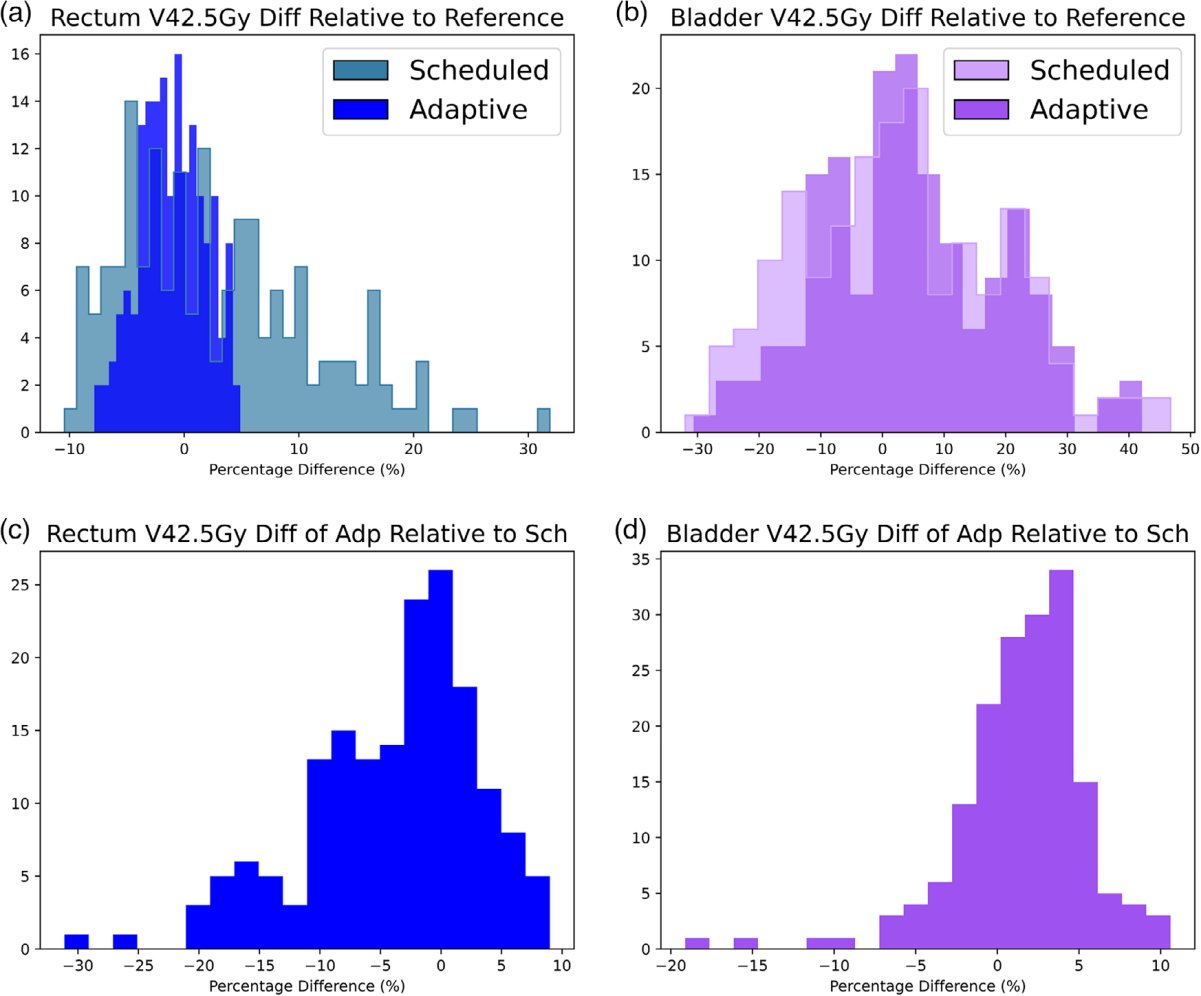
Distribution of dosimetric objective values from adaptive and scheduled treatment plans for the primary course of 20 patients treated to their prostatic fossa and pelvic lymph nodes. Panels (a) and (b) show the V42.5 to rectum and bladder of scheduled and adaptive treatment plans relative to the reference V42.5 value. Panels (c) and (d) show the relative difference between the V42.5 value of the adaptive versus the scheduled treatment plan for rectum and bladder, respectively.

An extension of the DVH distribution plotting allows users to slice through the various dosimetric objectives used for plan assessment (e.g., V40Gy to Rectum). Distributions can be overlaid or plotted side‐by‐side. Use of the MEDE gives an advantage over the intrinsic Ethos TPMS as these objectives can be adjusted and assessed post‐hoc, whereas the Ethos TPMS will only report objectives defined prior to authorizing treatment. The MEDE can also do direct comparisons between the Scheduled and Adaptive plan dose metrics. Example plots of these are presented in Figure 4.

## 4 DISCUSSION

Our MEDE software enables retrospective longitudinal assessment of oART patients with data that were not readily extracted from the Ethos TPMS without extensive time investment. Data mining improvements with the Ethos system are still in their nascent stage, with various early adopters developing independent methods for data collection and assessment.^4^ The MEDE software leverages the pre‐existing Mobius and Ethos architecture provided by the vendor to provide quick, effective longitudinal assessment for end users. Our visualization examples provide methods that demonstrate how the data are manipulated within the Pandas dataframe without extensive development of specialized knowledge or expertise. MEDE can be automated to extract data daily from an institutional Mobius database

A particular focus for the MEDE is to enable collaboration between oART programs. The patient indexing function has been designed to mimic how protocols assign patient IDs, with each institution identified by an NCI number and an incrementing patient identifier (e.g., 00019). Institutions may also be permitted by their IRB create an index to allow for retrospective lookback by the treating institution for data confirmation and retrospective outcome evaluation. Pandas dataframes are easily expandable and mergeable. This type of tool could allow for increased availability of adaptive data for trial review without transferring large datasets.

MEDE does suffer from several drawbacks, largely caused by a lack of detailed spatial data from both images and structure sets. Structure consistency checks are limited to center‐of‐mass position and volume and do not include more detailed volumetric assessment, such as Dice Similarity Coefficient or Hausdorff Distances from the adaptive session compared to reference. While this data are obtainable from the TPMS, we have shown the difference in time and data storage requirements which can be prohibitive depending on the size of the adaptive therapy practice. This tool is retrospective in nature and not able to rapidly extract this data for real‐time assessment that can inform clinician decision‐making during an adaptive therapy session.

## 5 CONCLUSION

We have developed a Python‐based data mining tool designed specifically to extract longitudinal data from oART patients treated with Ethos. This tool is designed to interact with the vendor‐provided environment with minimal institutional setup and provide anonymized data extraction that can be shared between participating institutions. Data visualization examples have been provided in an accompanying file that provides different methods for analyzing and presenting large adaptive datasets.

## AUTHOR CONTRIBUTIONS

Sean Tanny and Nicholas Sperling designed the project, acquired, and analyzed the data, and wrote the manuscript. Sean Tanny and Nicholas Sperling developed data the acquisition tool and revised the manuscript. Alexander Podgorsak made significant contribution to the design of the work and revised the manuscript. All authors approved the submitted draft and agreed to be accountable for all aspects of this work.

## CONFLICT OF INTEREST STATEMENT

The authors whose names are listed above certify that they have NO affiliations with or involvement in any organization or entity with any financial interest (such as honoraria; educational grants; participation in speakers’ bureaus; membership, employment, consultancies, stock ownership, or other equity interest; and expert testimony or patent‐licensing arrangements), or non‐financial interest (such as personal or professional relationships, affiliations, knowledge or beliefs) in the subject matter or materials discussed in this manuscript.
